# Malawian children with fast-breathing pneumonia with and without comorbidities

**DOI:** 10.1186/s41479-021-00081-y

**Published:** 2021-02-25

**Authors:** Amy Sarah Ginsburg, Tisungane Mvalo, Jun Hwang, Melda Phiri, Eric D. McCollum, Madalitso Maliwichi, Robert Schmicker, Ajib Phiri, Norman Lufesi, Susanne May

**Affiliations:** 1 University of Washington Clinical Trial Center, Building 29, Suite 250, 6200 NE 74th Street, Seattle, WA 98115 USA; 2 University of North Carolina Project, Lilongwe Medical Relief Fund Trust, Lilongwe, Malawi; 3 Johns Hopkins School of Medicine, Baltimore, MD USA; 4 College of Medicine, University of Malawi, Blantyre, Malawi; 5 Malawi Ministry of Health, Lilongwe, Malawi

**Keywords:** Fast-breathing, Community-acquired pneumonia, Comorbidity, Africa

## Abstract

**Background:**

Due to high risk of mortality, children with comorbidities are typically excluded from trials evaluating pneumonia treatment. Understanding heterogeneity of outcomes among children with pneumonia and comorbidities is critical to ensuring appropriate treatment.

**Methods:**

We explored whether the percentage of children with fast-breathing pneumonia cured at Day 14 was lower among those with selected comorbidities enrolled in a prospective observational study than among those enrolled in a concurrent randomized controlled trial evaluating treatment with amoxicillin in Lilongwe, Malawi.

**Results:**

Among 79 children with fast-breathing pneumonia in the prospective observational cohort, 57 (72.2%) had HIV infection/exposure, 20 (25.3%) had malaria, 2 (2.5%) had severe acute malnutrition, and 17 (21.5%) had anemia. Treatment failure rate was slightly (not significantly) lower in children with comorbidities (4.1%, 3/73) compared to those without comorbidities (4.5%, 25/552) similarly treated. There was no significant difference in clinical cure rates by Day 14 (95.8% with vs 96.7% without comorbidity).

**Conclusions:**

Children with fast-breathing pneumonia excluded from a concurrent clinical trial due to comorbidities did not fare worse. Children at higher risk whose caregivers seek care early and who receive appropriate risk assessment (e.g., pulse oximetry, hemoglobin, HIV/malaria testing) and treatment, can achieve clinical cure by Day 14.

**Trial registration:**

ClinicalTrials.govNCT02960919; registered November 8, 2016.

**Supplementary Information:**

The online version contains supplementary material available at 10.1186/s41479-021-00081-y.

## Background

**Fig. 1:**
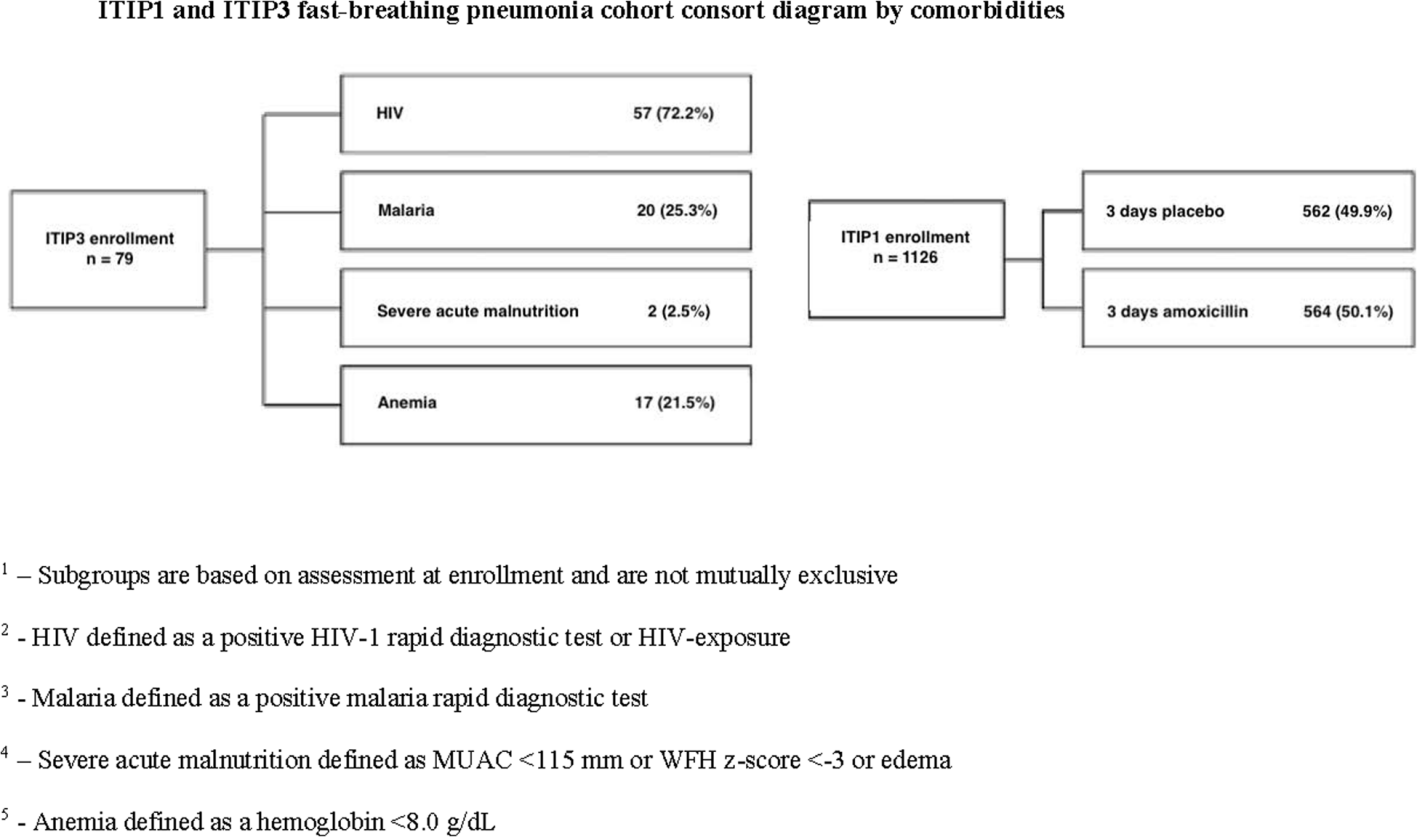
ITIP1 and ITIP3 fast-breathing pneumonia cohort consort diagram by comorbidities

Pneumonia is the leading infectious cause of childhood mortality worldwide [1]. As part of the Innovative Treatments in Pneumonia (ITIP) project in Lilongwe, Malawi, a randomized controlled clinical trial (ITIP1) was conducted to evaluate treatment with amoxicillin for fast-breathing pneumonia (FBP) in children (Table 1) [2]. Clinical trials evaluating treatment for pneumonia may exclude children with comorbidities who are at high risk for mortality or have other complications. Pneumonia in children with comorbidities is common, and many factors determine whether contact with an etiologic agent will result in a severe episode of pneumonia, and whether the episode will be deadly [3–7]. These factors can be related to the child (e.g., sex, age, underlying diseases), to the disease (e.g., type of infection), to the environment, the family and its socio-economic status, and to the health system and type of care [8]. A systematic review and meta-analysis of risk factors for mortality from acute lower respiratory infections (ALRI) in children under five years of age in low- and middle-income countries found chronic underlying diseases (odds ratio 4.76, 95% confidence interval 3.27–6.93), HIV/AIDS (4.68, 3.72–5.90), and severe malnutrition (4.27, 3.47–5.25) were associated with ALRI mortality [8]. In an effort to better understand pneumonia treatment outcomes among higher risk African children post-introduction of *Haemophilus influenzae* type b and *Streptococcus pneumoniae* conjugate vaccines, and to interpret the results of the concurrent FBP clinical trial (ITIP1), a prospective observational study (ITIP3) was conducted to assess the clinical outcomes of children aged 2 to 59 months with both pneumonia and comorbidities [2, 9]. We compare clinical outcomes of children with FBP and selected comorbidities (ITIP3) to those without these selected comorbidities (ITIP1) (Fig. 1).

**Table 1:** Study definitions, eligibility criteria, and clinical outcomes

**Study definitions**	
Fast-breathing pneumonia	Cough less than 14 days or difficulty breathing AND fast breathing for age
Chest-indrawing pneumonia	Cough less than 14 days or difficulty breathing AND visible indrawing of the chest wall with or without fast breathing for age
Fast breathing for age	Respiratory rate ≥ 50 breaths per minute (for children 2 to < 12 months of age) or ≥ 40 breaths per minute (for children ≥12 months of age)
Very fast breathing for age	≥70 breaths per minute (for children 2 to < 12 months of age) or ≥ 60 breaths per minute (for children ≥12 months of age)
Severe respiratory distress	Grunting, nasal flaring, and/or head nodding
Hypoxemia	Transcutaneous peripheral oxyhemoglobin saturation (SpO_2_) < 90% in room air, as assessed non-invasively by a pulse oximeter
World Health Organization (WHO) Integrated Management of Childhood Illness (IMCI) general danger signs	Lethargy or unconsciousness, convulsions, vomiting everything, inability to drink or breastfeed
Severe acute malnutrition	Weight for height/length < −3 SD, mid-upper arm circumference (MUAC) < 11.5 cm, or edema
HIV exposure	Children < 24 months of age with a HIV-infected mother
**ITIP1 and ITIP3 eligibility criteria**	
Inclusion criteria	• 2–59 months of age
	• Cough < 14 days or difficulty breathing
	• Fast breathing for age
	• ITIP3 fast-breathing pneumonia cohort: excluded from enrollment in ITIP1 clinical trial due to the presence of any of the following: ▪ Severe respiratory distress ▪ Hypoxemia ▪ Hemoglobin < 8.0 g/dL, if a positive malaria rapid diagnostic test (mRDT) ▪ Severe acute malnutrition ▪ Severe malaria (i.e., positive mRDT with any WHO IMCI general danger sign, stiff neck, abnormal bleeding, clinical jaundice, or hemoglobinuria) ▪ HIV seropositivity or HIV exposure
	• Ability and willingness of child’s caregiver to provide informed consent and to be available for follow-up for the planned duration of the study, including accepting a home visit if he/she fails to return for a scheduled study follow-up visit
Exclusion criteria	• Chest indrawing
	• Stridor when calm
	• Resolution of fast breathing after bronchodilator challenge (trial of rapid-acting inhaled bronchodilator for up to 3 times, 15–20 min apart) among those with audible or auscultatory wheeze at screening
	• Possible tuberculosis (coughing for more than 14 days)
	• Hemoglobin < 8.0 g/dL, if a negative mRDT
	• Known allergy to penicillin or amoxicillin
	• Receipt of an antibiotic treatment in the 48 h prior to the study
	• Living outside the study area
	• Any medical or psychosocial condition or circumstance that, in the opinion of the investigators, would interfere with the conduct of the study or for which study participation might jeopardize the child’s health
	• Participation in a clinical study of an investigational product within 12 weeks prior to enrollment or planning to begin participation during this study
	• Prior participation in any ITIP study during a previous pneumonia diagnosis
	• ITIP1 cohort only: ▪ Severe respiratory distress ▪ Hypoxemia ▪ Hemoglobin < 8.0 g/dL, if a positive mRDT ▪ Severe acute malnutrition ▪ Severe malaria (i.e., positive mRDT with any WHO IMCI general danger sign, stiff neck, abnormal bleeding, clinical jaundice, or hemoglobinuria) ▪ HIV seropositivity or HIV exposure
**ITIP1 and ITIP3 clinical outcomes**	
Treatment failure^a^	For both ITIP1 and ITIP3, development of very fast breathing, chest indrawing, severe respiratory distress, hypoxemia, WHO IMCI general danger signs, fever, or change in antibiotics:
	• For ITIP1, prior to or on Day 4 (i.e., anytime between Days 2, 3, or 4)
	• For ITIP3, on Day 6 only
	For ITIP1 only, missing two or more doses due to vomiting, or hospitalization due to pneumonia (including prolonged hospitalization or re-admission) prior to or on Day 4
	Death:
	• For ITIP1, prior to or on Day 4
	• For ITIP3, prior to or on Day 6
Clinically cured^b^	For both ITIP1 and ITIP3, absence of fast-breathing pneumonia, very fast breathing for age, chest indrawing, severe respiratory distress, hypoxemia, WHO IMCI general danger signs, and fever by Day 14:
	• Cured but failed initial antibiotic treatment regimen
	• Cured and did not fail initial antibiotic treatment regimen
Not clinically cured^b^	Day 14:
	• Deteriorating
	• Stable (not improving or deteriorating, prognosis unclear)
	On or prior to Day 16:
	• Death

^a^Children with treatment failure in ITIP1 may have had their Day 4 visit 1 day later (on Day 5) and children with treatment failure in ITIP3 may have had their Day 6 visit 1 day later (on Day 7)

^b^Clinically cured status is assessed from the last visit that may have occurred 2 days earlier to 2 days later than the scheduled day (i.e., on actual Days 12 through 16)

## Methods

### Study design

The objective of this study was to investigate whether the percentage of children with FBP cured at Day 14 was lower among those with HIV infection or exposure, malaria, severe acute malnutrition, or anemia than among those without these selected comorbidities who were treated similarly. Children aged 2 to 59 months meeting the case definition of FBP in the pediatric outpatient departments of Kamuzu Central Hospital (KCH) and Bwaila District Hospital (BDH) in Lilongwe, Malawi were screened by study staff to determine ITIP eligibility (Table 1). Those who were excluded from the ITIP1 clinical trial because of comorbidities were assessed for enrollment into ITIP3. ITIP1 enrollment began June 7, 2016 with the last visit completed June 20, 2017. A total of 1126 children were enrolled in ITIP1 with 564 randomized to receive 3 days of oral amoxicillin, the standard of care treatment group, and 562 randomized to receive placebo [2]. ITIP3 FBP enrollment began November 8, 2016 with the last study visit completed on July 14, 2018. Per Malawian guidelines and KCH protocols, each child in the ITIP3 FBP cohort received standard of care treatment for their pneumonia, typically 5 days of oral amoxicillin, and for their comorbidities.

The study was conducted in accordance with the International Conference on Harmonisation, Good Clinical Practice and the Declaration of Helsinki 2008, and was approved by the Western Institutional Review Board in the state of Washington, USA; the College of Medicine Research and Ethics Committee, Blantyre, Malawi; and the Malawi Pharmacy, Medicines and Poisons Board. The study was registered with ClinicalTrials.gov: NCT02960919.

### Study procedures

On Day 1, after enrollment informed consent was obtained from the caregiver or legal guardian by study staff, ITIP eligible children received a physical examination, and information regarding their medical and vaccination history along with additional socio-demographic information was collected. Screening and enrollment were conducted in the pediatric outpatient departments of KCH or BDH, with BDH enrollees transferred to KCH for continued evaluation, observation, and admission, if indicated. Hospital observation or admission and all follow-up visits occurred solely at KCH.

Enrolled children were treated and prospectively followed by study staff with scheduled study visits on Days 2 to 4 and 14 (all in-person) in ITIP1 and on Days 2 (if hospitalized), 6 and 14 (in-person), and 30 (by phone) in ITIP3. During in-person follow-up visits, study staff reviewed the medical history since the last study visit and performed a physical examination including respiratory rate, chest indrawing, and pulse oximetry assessments. In case of a no-show at scheduled follow-up visits, a home visit was conducted by study staff. If a phone call on Day 30 was not possible due to no phone in the home, study staff conducted a home visit to obtain Day 30 outcome information in ITIP3. More detailed study procedures for children enrolled in ITIP1 and ITIP3 have been described previously [2, 9].

### Study outcomes

Primary endpoints for this study analysis included treatment failure (TF) rates and clinical outcomes of children treated for FBP and clinical cure rates at Day 14 in select subgroups defined by comorbidities (HIV infection or exposure, malaria, severe acute malnutrition, or anemia) with comparisons to children in the standard of care treatment group of ITIP1. Secondary endpoints included: treatment responses (vital signs, oxygen saturation, length of hospital stay); and proportion of children who were re-hospitalized or died.

Time points for TF assessment were chosen in accordance with current World Health Organization (WHO) pneumonia management guidelines [10]. In ITIP3, TF was assessed on Day 6 and in ITIP1, TF was assessed on or prior to Day 4. Children enrolled to ITIP3 were followed for 30 days post-enrollment. ITIP1’s clinical trial population was followed for 14 days post-enrollment. Of note, development of very fast breathing (as defined in Table 1) is included here in the TF definition for ITIP1 and ITIP3, but was not included in the TF definition for ITIP1 protocol and primary manuscript [2].

### Statistical analysis

Descriptive statistics are provided for clinical outcomes. Chi-squared statistics or Fisher’s exact tests were used to compare TF and clinically cured rates between ITIP3 and ITIP1 treatment groups. Tests were performed as two-sided tests with alpha = 0.05. No adjustments were made for confounders, and no formal statistical comparisons were made by comorbidity subgroup due to the small number of ITIP3 FBP children. No adjustments were made for multiple comparisons because of the observational and exploratory nature of this study.

## Results

Of the 79 ITIP3 FBP children enrolled, 72.2% (57/79) were HIV-infected or exposed, 25.3% (20/79) had malaria, 2.5% (2/79) met severe acute malnutrition criteria either by mid-upper arm circumference < 115 mm or weight-for-height z-score < − 3, and 21.5% (17/79) had anemia defined as a hemoglobin < 8.0 g/dL (Fig. 1). ITIP3 TF assessment data and clinical outcome by Day 14 were available for 71 (89.9%) children. ITIP1 TF assessment data by Day 4 was available for 552 and 543 children in the amoxicillin and placebo groups, respectively, and ITIP1 clinical outcome data by Day 14 was available for 543 and 527 children in the amoxicillin and placebo groups, respectively.

When comparing ITIP3’s FBP cohort to all children enrolled in ITIP1’s FBP clinical trial, there were a few differences in baseline characteristics (Table 2). Children with FBP in ITIP3 were significantly younger than those in ITIP1, with 54.4% (43/79) children 2–11 months of age compared to 34.8% (392/1126) in ITIP1 (*p* < 0.01). When examining fast breathing for age, more children in ITIP3 than in ITIP1 met very fast breathing criteria. Among those less than 12 months of age, 4.7% (2/43) had very fast breathing in ITIP3’s FBP cohort compared to 2.6% (10/392) in ITIP1 (*p* = 0.34). Among those older than 12 months of age, 22.2% (8/36) had very fast breathing in ITIP3’s FBP cohort compared to 4.4% (32/734) in ITIP1 (*p* < 0.01). There was a non-significantly higher percentage of fevers in ITIP3’s FBP cohort (39.2%, 31/79) than in ITIP1 (29.8%, 335/1126; *p* = 0.08).

**Table 2:** ITIP3 fast-breathing pneumonia cohort and ITIP1 baseline characteristics

	ITIP3 fast-breathing pneumonia cohort					ITIP1
	HIV^a,b^	Malaria^a,c^	Severe acute malnutrition^a,e^	Anemia^a,d^	Total	
	(***n*** = 57)	(***n*** = 20)	(***n*** = 2)	(***n*** = 17)	(***n*** = 79)	(***n*** = 1126)
Age (months)						
Median (IQR)	9 (5, 18)	19 (11, 24)	10 (10, 11)	18 (11, 24)	11 (6, 19)	18 (8, 32)
2–6, n (%)	20 (35.1)	2 (10.0)	0 (0.0)	1 (5.9)	22 (27.8)	222 (19.7)
7–11, n (%)	13 (22.8)	5 (25.0)	2 (100.0)	5 (29.4)	21 (26.6)	170 (15.1)
12–23, n (%)	21 (36.8)	6 (30.0)	0 (0.0)	5 (29.4)	25 (31.6)	294 (26.1)
24–35, n (%)	1 (1.8)	4 (20.0)	0 (0.0)	3 (17.6)	5 (6.3)	215 (19.1)
36–59, n (%)	2 (3.5)	3 (15.0)	0 (0.0)	3 (17.6)	6 (7.6)	225 (20.0)
Sex						
Male, n (%)	28 (49.1)	12 (60.0)	1 (50.0)	9 (52.9)	40 (50.6)	525 (46.6)
Female, n (%)	29 (50.9)	8 (40.0)	1 (50.0)	8 (47.1)	39 (49.4)	601 (53.4)
Respiratory rate^f^ (breaths/minute)						
Age < 12 months						
< 50, n (%)	1 (3.0)	0 (0.0)	1 (50.0)	0 (0.0)	2 (4.7)	1 (0.3)
50–59, n (%)	21 (63.6)	3 (429)	1 (50.0)	3 (50.0)	26 (60.5)	279 (71.2)
60–69, n (%)	11 (33.3)	2 (28.6)	0 (0.0)	2 (33.3)	13 (30.2)	102 (26)
≥ 70, n (%)	0 (0.0)	2 (28.6)	0 (0.0)	1 (16.7)	2 (4.7)	10 (2.6)
Age ≥ 12 months						
< 40, n (%)	0 (0.0)	0 (0.0)	0 (0.0)	0 (0.0)	0 (0.0)	0 (0.0)
40–49, n (%)	12 (50.0)	4 (30.8)	0 (0.0)	3 (27.3)	17 (47.2)	467 (63.6)
50–59, n (%)	8 (33.3)	3 (23.1)	0 (0.0)	2 (18.2)	11 (30.6)	235 (32)
≥ 60, n (%)	4 (16.7)	6 (46.2)	0 (0.0)	6 (54.5)	8 (22.2)	32 (4.4)
Oxygen saturation^g^ (%)						
< 90, n (%)	0 (0.0)	0 (0.0)	0 (0.0)	0 (0.0)	0 (0.0)	0 (0.0)
90–92, n (%)	1 (1.8)	0 (0.0)	0 (0.0)	0 (0.0)	1 (1.3)	0 (0.0)
≥ 93, n (%)	56 (98.2)	20 (100.0)	2 (100.0)	17 (100.0)	78 (98.7)	1126 (100.0)
Axillary temperature^f^ (°C)						
≥ 38, n (%)	16 (28.1)	16 (80.0)	1 (50.0)	14 (82.4)	31 (39.2)	335 (29.8)
< 38, n (%)	41 (71.9)	4 (20.0)	1 (50.0)	3 (17.6)	48 (60.8)	791 (70.2)
Heart rate^f^ (beats/minute)						
Median	146	159	148	160	151	147
Minimum, Maximum	117, 179	141, 192	145, 152	141, 191	117, 192	101, 196

^a^Subgroup based on data from screening/enrollment form; subgroups are not mutually exclusive

^b^HIV defined as a positive HIV-1 rapid diagnostic test or HIV exposure

^c^Malaria defined as a positive malaria rapid diagnostic test

^d^Anemia defined as a hemoglobin < 8.0 g/dL

^e^Severe acute malnutrition defined as MUAC < 115 mm or WFH z-score < −3

^f^Maximum value between screening and enrollment

^g^ Minimum value between screening and enrollment

A slightly higher percent of children in ITIP1 received age-appropriate numbers of pentavalent and pneumococcal conjugate vaccines than in ITIP3’s FBP cohort (54.4% in ITIP3 for both vaccines versus 57.6% for pentavalent vaccine (*p* = 0.58) and 57.5% for pneumococcal conjugate vaccine (*p* = 0.59) in ITIP1, Appendix S1). Overall hospitalizations were higher in ITIP3’s FBP cohort (11.4%, 9/79) compared to ITIP1 (8.7%, 98/1126), but the difference was not statistically significant (*p* = 0.54, Appendix S2). One child was re-hospitalized in ITIP3 and four children were re-hospitalized in ITIP1 (data not shown).

In ITIP3’s FBP cohort, TF was 4.1% (3/73) compared to 4.5% (25/552) in ITIP1’s amoxicillin treatment group (*p* > 0.99) (Table 3). In contrast, 7.6% (41/543) of children in ITIP1’s placebo group had TF when compared to ITIP3’s FBP cohort (*p* = 0.28). There were no significant differences in rates of those clinically cured by Day 14 between ITIP3’s FBP cohort (95.8%) and either ITIP1’s amoxicillin (96.7%; *p* = 0.73) or ITIP1’s placebo (98.1%; *p* = 0.19) groups (Table 4). There were no deaths in ITIP3’s FBP cohort by the Day14 assessment nor in ITIP1’s clinical trial (over the entire observation period). Post-Day 14, there were 2 deaths recorded in ITIP3’s FBP cohort, one child with danger sign pneumonia and another child with pulmonary tuberculosis and severe acute malnutrition. The child with danger sign pneumonia who died was clinically cured based on clinical data on Day 14 but died on Day 22. The child with pulmonary tuberculosis and severe acute malnutrition was clinically not cured based on clinical data on Day 14. There was no follow-up (including assessment of deaths) in ITIP1’s clinical trial post-Day 14.

**Table 3:** ITIP3 fast-breathing pneumonia cohort and ITIP1 treatment failure rates^a^

	ITIP3 fast-breathing pneumonia cohort					ITIP1	
	HIV^**b,c**^	Malaria^**b,d**^	Severe acute manlnutrition^**b,f**^	Anemia^**b,e**^	Total	3 days placebo	3 days amoxicillin
	(*n* = 54)	(*n* = 17)	(*n* = 2)	(*n* = 14)	(*n* = 73)	(*n* = 543)	(*n* = 552)
Treatment failure^g^	1 (1.9%)	1 (5.9%)	0 (0.0%)	1 (7.1%)	3 (4.1%)	41 (7.6%)	25 (4.5%)
WHO IMCI general danger^g^ signs	0 (0.0%)	0 (0.0%)	0 (0.0%)	0 (0.0%)	0 (0.0%)	3 (0.6%)	5 (0.9%)
Severe respiratory distress^g^	0 (0.0%)	0 (0.0%)	0 (0.0%)	0 (0.0%)	0 (0.0%)	6 (1.1%)	0 (0.0%)
Hypoxemia^g^	0 (0.0%)	0 (0.0%)	0 (0.0%)	0 (0.0%)	0 (0.0%)	0 (0.0%)	0 (0.0%)
Chest indrawing^g^	1 (1.9%)	0 (0.0%)	0 (0.0%)	0 (0.0%)	1 (1.4%)	15 (2.8%)	10 (1.8%)
Very fast breathing^g^	0 (0.0%)	0 (0.0%)	0 (0.0%)	0 (0.0%)	0 (0.0%)	3 (0.6%)	3 (0.5%)
Fever^g^	0 (0.0%)	1 (5.9%)	0 (0.0%)	1 (7.1%)	1 (1.4%)	15 (2.8%)	2 (0.4%)
Death^h^	0 (0.0%)	0 (0.0%)	0 (0.0%)	0 (0.0%)	0 (0.0%)	0 (0.0%)	0 (0.0%)
Change in antibiotic^g^	1 (1.9%)	1 (5.9%)	0 (0.0%)	1 (7.1%)	3 (4.1%)	37 (6.8%)	21 (3.8%)

^a^Treatment failure rates are a percentage of those with a known treatment failure status

^b^Subgroup based on data from screening/enrollment; subgroups are not mutually exclusive

^c^HIV defined as a positive HIV-1 rapid diagnostic test or HIV-exposed

^d^Malaria defined as a positive malaria rapid diagnostic test

^e^Anemia defined as a hemoglobin < 8.0 g/dL

^f^Severe acute malnutrition defined as MUAC < 115 mm or WFH z-score < −3

^g^On Day 6 for ITIP3; on or prior to Day 4 for ITIP1

^h^On or prior to Day 6 for ITIP3; on or prior to Day 4 for ITIP1

**Table 4:** ITIP3 fast-breathing pneumonia cohort and ITIP1 clinical cure rates^a^

	ITIP3 fast-breathing pneumonia cohort					ITIP1	
	HIV^b,c^	Malaria^b,d^	Severe acute malnutrition^b,f^	Anemia^b,e^	Total	3 days placebo	3 days amoxicillin
	(*N* = 57)	(*N* = 20)	(*N* = 2)	(*N* = 17)	(*N* = 79)	(*N* = 562)	(*N* = 564)
**Day 14 clinical outcome**							
Cured	50 / 51 (98%)	15 / 17 (88.2%)	2 / 2 (100%)	13 / 14 (92.9%)	68 / 71 (95.8%)	517 / 527 (98.1%)	525 / 543 (96.7%)
Not cured	1 / 51 (2%)	2 / 17 (11.8%)	0 / 2 (0%)	1 / 14 (7.1%)	3 / 71 (4.2%)	10 / 527 (1.9%)	18 / 543 (3.3%)
Unknown clinical outcome	6 / 57 (10.5%)	3 / 20 (15%)	0 / 2 (0%)	3 / 17 (17.6%)	8 / 79 (10.1%)	35 / 562 (6.2%)	21 / 564 (3.7%)
Loss to follow-up or consent withdrawn	3 / 57 (5.3%)	3 / 20 (15%)	0 / 2 (0%)	3 / 17 (17.6%)	5 / 79 (6.3%)	27 / 562 (4.8%)	18 / 564 (3.2%)
Missing visit	3 / 57 (5.3%)	0 / 20 (0%)	0 / 2 (0%)	0 / 17 (0%)	3 / 79 (3.8%)	8 / 562 (1.4%)	3 / 564 (0.5%)

^a^Cured and not cured rates are a percentage of those with a known clinical outcome status; unknown rates are a percentage of total

^b^Subgroup based on data from screening/enrollment; subgroups are not mutually exclusive

^c^HIV defined as a positive HIV-1 rapid diagnostic test or HIV-exposed

^d^Malaria defined as a positive malaria rapid diagnostic test

^e^Anemia defined as a hemoglobin < 8.0 g/dL

^f^Severe acute malnutrition defined as MUAC < 115 mm or WFH z-score < −3

## Discussion

Many children with pneumonia have comorbidities, and these children can be at high risk for pneumonia mortality. In our ITIP3 study population, the number of children with FBP and one or more comorbidities was low (*n* = 79), with HIV and malaria the most common comorbidities. When comparing children in ITIP3’s FBP cohort to those in our ITIP1 FBP clinical trial, TF by Day 6 was not significantly different among children in ITIP3; and neither were clinical cure rates by Day 14. Children with FBP who were excluded from the concurrent ITIP1 clinical trial due to comorbidities did not fare worse in our prospective observational study. Because this current analysis focused only on children with FBP rather than more severe pneumonia, it is likely that we were able to identify children early in their disease course. Another possibility is that because fast breathing is a non-specific sign, some proportion of these children had a primary process other than pneumonia.

These results may be explained in part by the systematic and comprehensive risk assessment done at the first point of care for study participants. For example, while HIV is a significant risk factor for mortality, HIV screening is inconsistently done in practice [11–13]. However, in ITIP3, children were systematically tested for HIV such that those with HIV were identified promptly at the first point of care. While such children still received less structured care consistent with real-world clinical management, outcomes were comparable to those of lower risk children without comorbidities in ITIP1 who received more structured care and closer follow-up typical of a clinical trial. Additionally, other signs of illness such as fever and difficult breathing were identified early among these children, with many of them receiving care within 2 to 3 days of initial sign of illness. These results may suggest that if higher risk children seek care early, and risk is systematically and appropriately assessed (e.g., pulse oximetry, hemoglobin, HIV and malaria testing) at the first point of care, nearly all can achieve clinical cure by Day 14, similar to those without comorbidities [4, 14, 15].

One important caveat in this study is that most of the study population was recruited from an outpatient clinic at BDH whose catchment area is urban or peri-urban Lilongwe, and we were not able to account for children who went directly to KCH, were referred to KCH from a rural health center, or did not access care. Thus, there may have been selection bias if children with more severe cases of pneumonia bypassed BDH. Another limitation included enrollment to ITIP3’s FBP cohort being curtailed when ITIP1’s FBP trial stopped enrolling. This led to enrollment of only a small number of children with FBP, resulting in high uncertainty around estimates which was further compounded by missing outcome data. These small numbers may limit generalizability to all children with comorbidities; they also may reflect that children with pneumonia and comorbidities may progress to more severe disease faster.

We also focused on selected common, major comorbidities, and not all potential comorbidities, such as a history of being born preterm or low birthweight. Other limitations were different treatment regimens used, outcome assessments at different timepoints, and the lack of structured follow-up in ITIP3 compared to ITIP1 (study visits were more frequent for ITIP1 than ITIP3 during the first 14 days), which limited the information available regarding clinical course and made direct comparisons difficult. These structural differences were intentional because ITIP1 was a clinical trial with randomized treatments and close follow-up and ITIP3 was an observational study using standard of care treatments with some additional follow-up. We applied the relevant WHO management guidelines and timepoints for TF assessment specific to each ITIP1 and ITIP3 group. We were unable to apply the same TF criteria to both groups given the guidelines themselves are different. We chose to apply WHO guidelines for diagnosing and treating pneumonia to these groups because it was important for our results to be representative of real-world care, and these are the guidelines that healthcare providers use during routine care conditions. Finally, this is a complete case analysis and other more extensive sensitivity analyses were not performed due to the exploratory and observational nature of the study.

## Conclusions

In conclusion, clinical cure rates by Day 14 were high in ITIP3’s FBP cohort. Clinical cure rates by Day 14 were similar between ITIP3 children excluded from ITIP1 and those in ITIP1 treated similarly. These children with FBP were not at high risk for mortality despite having comorbidities. If risk factors are promptly identified and relevant clinical guidelines are followed, overall outcomes among children with FBP and comorbidities can be comparable to those among low-risk children with FBP and no comorbidities. Systematic risk assessment coupled with appropriate management and referral is effective in limiting adverse outcomes in children with FBP and comorbidities in this setting.

## Supplementary Information

**Additional file 1: Appendix S1**. ITIP3 fast-breathing pneumonia cohort and ITIP1 pentavalent and pneumococcal conjugate vaccinations. **Appendix S2**. ITIP3 fast-breathing pneumonia cohort and ITIP1 hospitalizations and lengths of stay during study participation

## Data Availability

Upon request, deidentified participant data that underlie the results reported in this article will be made available to researchers who provide a methodologically sound proposal following publication of the planned primary and secondary analyses. Proposals should be directed to the corresponding author.

## Abbreviations

ALRI: Acute lower respiratory infections
BDH: Bwaila District Hospital
FBP: Fast-breathing pneumonia
ITIP: Innovative Treatments in Pneumonia
IMCI: Integrated Management of Childhood Illness
KCH: Kamuzu Central Hospital
TF: Treatment failure
WHO: World Health Organization
