# Supplementary material for: Malawian children with fast-breathing pneumonia with and without comorbidities
Source: Pneumonia (Nathan). 2021 Feb 25;13:3. doi: 10.1186/s41479-021-00081-y (PMC7905626; doi:10.1186/s41479-021-00081-y)
Supplement: Supplementary file 1 — Additional file 1: Appendix S1. ITIP3 fast-breathing pneumonia cohort and ITIP1 pentavalent and pneumococcal conjugate vaccinations. Appendix S2. ITIP3 fast-breathing pneumonia cohort and ITIP1 hospitalizations and lengths of stay during study participation [file 41479_2021_81_MOESM1_ESM.pdf]

Appendix S1. ITIP3 fast-breathing pneumonia cohort and ITIP1 pentavalent and pneumococcal conjugate vaccinations

	ITIP3 fast-breathing pneumonia cohort	ITIP1
	(n=79)	(n=1126)
Pentavalent vaccine		
Received age-appropriate number of doses,[1] n (%)	43 (54.4%)	649 (57.6%)
All doses unknown, n (%)	30 (38%)	412 (36.6%)
Some doses missed or unknown, n (%)	6 (7.6%)	65 (5.8%)
Received 2 doses, n (%)	5 (6.3%)	45 (4%)
Received 1 dose, n (%)	1 (1.3%)	17 (1.5%)
Received 0 doses, n (%)	0 (0%)	3 (0.3%)
Pneumococcal conjugate vaccine		
Received age-appropriate number of doses,[1] n (%)	43 (54.4%)	648 (57.5%)
All doses unknown, n (%)	30 (38%)	413 (36.7%)
Some doses missed or unknown, n (%)	6 (7.6%)	65 (5.8%)

Received 2 doses, n (%)	5 (6.3%)	46 (4.1%)
Received 1 dose, n (%)	1 (1.3%)	16 (1.4%)
Received 0 doses, n (%)	0 (0%)	3 (0.3%)

[1] - One dose if at least 6 weeks and up to 10 weeks of age, two doses if at least 10 weeks and up to 14 weeks of age, three doses if at least 14 weeks of age

Appendix S2. ITIP3 fast-breathing pneumonia cohort and ITIP1 hospitalizations and lengths of stay during study participation

	ITIP3 fast-breathing pneumonia cohort	ITIP1
	**(n=79)**	**(n=1126)**
Hospitalized		
0 times, n (%)	70 (88.6)	1028 (91.3)
1 time, n (%)	8 (10.1)	94 (8.3)
2 times, n (%)	1 (1.3)	4 (0.4)
3 times, n (%)	0 (0.0)	0 (0.0)
Primary reason hospitalized		
Pneumonia with WHO IMCI general danger signs or severe respiratory distress		
n (% of times hospitalized)	5 (50.0)	78 (76.5)
Mean (sd) length of stay (days)	2.5 (1.4)	3.2 (2.9)

Median (IQR) length of stay (days)	2.0 (0.2)	3.0 (1.0)
WHO IMCI general danger signs		
n (% of times hospitalized)	0 (0.0)	5 (4.9)
Mean (sd) length of stay (days)	--	3.2 (1.9)
Median (IQR) length of stay (days)	--	3.0 (2.0)
Malaria		
n (% of times hospitalized)	1 (10.0)	3 (2.9)
Mean (sd) length of stay (days)	1.9 (--)	1.7 (1.2)
Median (IQR) length of stay (days)	1.9 (0.0)	1.0 (1.0)
Other		
n (% of times hospitalized)	4 (40.0)	17 (16.7)
Mean (sd) length of stay (days)	4.0 (3.5)	2.8 (1.3)
Median (IQR) length of stay (days)	3.5 (5.3)	2.0 (2.0)
